# Characterizing the Retinal Phenotype in the High-Fat Diet and Western Diet Mouse Models of Prediabetes

**DOI:** 10.3390/cells9020464

**Published:** 2020-02-18

**Authors:** Bright Asare-Bediako, Sunil K. Noothi, Sergio Li Calzi, Baskaran Athmanathan, Cristiano P. Vieira, Yvonne Adu-Agyeiwaah, Mariana Dupont, Bryce A. Jones, Xiaoxin X. Wang, Dibyendu Chakraborty, Moshe Levi, Prabhakara R. Nagareddy, Maria B. Grant

**Affiliations:** 1 Vision Science Graduate Program, School of Optometry, University of Alabama at Birmingham, Birmingham, AL 35233, USA; basareb@uab.edu (B.A.-B.); yvonnad@uab.edu (Y.A.-A.); mdupont@uab.edu (M.D.); 2 Department of Ophthalmology and Visual Sciences, School of Medicine, The University of Alabama at Birmingham, Birmingham, AL 35294, USA; sunilnooti@uabmc.edu (S.K.N.); scalzi@uabmc.edu (S.L.C.); cvieira@uabmc.edu (C.P.V.); dchakraborty@uabmc.edu (D.C.); 3 Division of Cardiac Surgery, Department of Surgery, Ohio State University Wexner Medical Center, Columbus, OH 43210, USA; baskaran.athmanathan@osumc.edu (B.A.); prabhakara.nagareddy@osumc.edu (P.R.N.); 4 Department of Pharmacology and Physiology, Georgetown University, Washington, DC 20057, USA; baj46@georgetown.edu; 5 Department of Biochemistry and Molecular & Cellular Biology, Georgetown University, Washington, DC 20057, USA; xiaoxin.wang@georgetown.edu (X.X.W.); moshe.levi@georgetown.edu (M.L.)

**Keywords:** retinal phenotype, neural infarcts, vascular leakage

## Abstract

We sought to delineate the retinal features associated with the high-fat diet (HFD) mouse, a widely used model of obesity. C57BL/6 mice were fed either a high-fat (60% fat; HFD) or low-fat (10% fat; LFD) diet for up to 12 months. The effect of HFD on body weight and insulin resistance were measured. The retina was assessed by electroretinogram (ERG), fundus photography, permeability studies, and trypsin digests for enumeration of acellular capillaries. The HFD cohort experienced hypercholesterolemia when compared to the LFD cohort, but not hyperglycemia. HFD mice developed a higher body weight (60.33 g vs. 30.17g, *p* < 0.0001) as well as a reduced insulin sensitivity index (9.418 vs. 62.01, *p* = 0.0002) compared to LFD controls. At 6 months, retinal functional testing demonstrated a reduction in a-wave and b-wave amplitudes. At 12 months, mice on HFD showed evidence of increased retinal nerve infarcts and vascular leakage, reduced vascular density, but no increase in number of acellular capillaries compared to LFD mice. In conclusion, the HFD mouse is a useful model for examining the effect of prediabetes and hypercholesterolemia on the retina. The HFD-induced changes appear to occur slower than those observed in type 2 diabetes (T2D) models but are consistent with other retinopathy models, showing neural damage prior to vascular changes.

## 1. Introduction

Diabetes is now considered a worldwide epidemic [1,2]. Recent reports indicate that over 90% of diabetic individuals have type 2 diabetes (T2D) [3,4]. The most common microvascular complication of diabetes is diabetic retinopathy (DR) [2]. Despite a growing number of different approaches to arrest DR, the incidence and prevalence of DR continues to rise [5]. The understanding of the pathogenesis of DR remains incomplete [4], and this is, in part, due to the lack of readily available models that completely recapitulate the metabolic phenotype [6]. The high-fat diet (HFD) mouse model has been described as a robust model for investigating obesity-associated T2D and its related metabolic complications [7]. Studies have shown that HFD-fed mice develop obesity, impaired glucose tolerance, and reduced insulin sensitivity [8,9] with systemic manifestations involving adipose tissue [10], liver [8], and kidneys [11]. However, the ocular changes associated with the HFD model have not been fully investigated. Moreover, the typical Western diet (WD; 40% fat) has also been given to rodents to recapitulate obesity-driven pathology. However, to mimic the features of T2D, the administration of low-dose Streptozotocin (STZ) is also given to the WD mice [12,13,14].

The retinal response to high fat exposure would likely involve local changes in the expression of lipid transport proteins, such as the liver X receptors (LXRs). The LXRs are the key transcription factors that regulate lipid and cholesterol metabolism [15]. While liver X receptor alpha (LXRα) is expressed only in some tissues, the expression of liver X receptor beta (LXRβ) is ubiquitous [12]. Previously we showed that whole body LXRα/β deficiency resulted in the generation of increased numbers of acellular capillaries, while LXR agonists improved DR in Streptozotocin (STZ)-induced diabetes [12] and in diabetic Lepr^db/db^ (db/db) mice [16]; however, it is not known if the WD modulates the expression of LXR in the retina.

Retinopathy is typically characterized by macroglia activation and gliosis identified by glial fibrillary acidic protein (GFAP) overexpression, which can be considered as a marker for retinal damage [17,18]. In the healthy mammalian retina, GFAP is expressed only in astrocytes and not in Muller cells. Following inherited or acquired retinal pathology, GFAP is expressed also in Muller cells [19,20]. GFAP expression in Muller cells has been widely used as a cellular marker for retinal pathology [21,22,23,24,25]. Hypoxia-inducible factor 1 alpha (HIF-1α) is known to be a key regulator of a tissue’s response to hypoxia [26] and plays a role in obesity-induced metabolic syndrome. It has been shown that HFD leads to gradual increase in HIF-1α and associated pathological changes in the liver [27,28]. However, the role of HIF-1α in the retina of WD-fed mice is not known.

A better understanding of DR in obesity-driven models is needed and may facilitate the optimal choice of disease models for future investigations. Thus, in the present study, we hypothesized that HFD and WD feeding would result in a distinct retinal phenotype and a time course slower than that observed in models of T2D, such as the db/db mouse [29] or the high fructose and high fat fed mouse [30]. For this purpose, we characterized not only systemic endpoints of glucose and lipid metabolism but also the function of the retina and development of retinal pathology, including retinal vascular changes and changes in expression of the critical proteins LXRβ, HIF-1α, and GFAP.

## 2. Materials and Methods

### 2.1. Animals

All animal experiments were approved by the University of Alabama at Birmingham (IACUC-20467, approved on 06/16/2016) and Georgetown University (animal project #2017-0059, approved on 10/27/2017), and followed the Association for Research in Vision and Ophthalmology Statement for the Use of Animals. Six to eight-week-old C57BL/6J mice were fed either a low-fat diet (LFD) (10%kcal fat, 70%kcal carbohydrate, 20% protein), a Western diet (40% kcal fat, 43% kcal carbohydrate, 17%kcal protein), or a HFD (60% kcal fat, 20% kcal carbohydrate, 20% kcal protein) for up to 12 months. Diets were purchased from Research Diets, Inc, New Brunswick, NJ, USA. Full details of the composition of each diet is given in Supplementary Table S1.

### 2.2. Body Composition, Glucose Tolerance, and Insulin Sensitivity Testing

The fat mass, lean mass, and water content of the animals were measured by magnetic resonance imaging using EchoMRI (Echo Medical Systems, LLC, Houston, TX, USA). For glucose and insulin tolerance tests, mice were fasted for 5-6 h, injected intraperitoneally with D-glucose at 1.5 g/kg of lean mass and tail bled for glucose and insulin measurements. Blood glucose and insulin levels were measured 0, 15, 30, 45, 60, and 120 min after glucose administration. The insulin sensitivity index (ISI) was estimated using the Matsuda–Defronzo method [31].

### 2.3. Electroretinogram (ERG)

ERGs were performed using a LKC Bigshot ERG system. Briefly, mice were dark-adapted overnight. The animals were anesthetized with ketamine (80 mg/kg total body mass) and xylazine (15 mg/kg total body mass), then dilated with atropine/phenylephrine under dim red light. Once dilated, animals were exposed to 5 full-field white light flashes at 0.25 and 2.5 cd.s/m^2^ under scotopic conditions. The animals were then light-adapted for 5 min and exposed to 10–15 full-field white light flashes at 10 and 25 cd.s/m^2^ under photopic conditions. Responses were averaged and analyzed using the LKC EM software.

### 2.4. Fundus Photography and Fluorescein Angiography

Fundus photography and fluorescein angiography were performed using the Phoenix Micron IV retinal imaging microscope (Phoenix Technology Group, Pleasanton, CA, USA). Briefly, mice were anesthetized with ketamine and xylazine, then dilated with atropine/phenlylephrine, as described above. Once dilated, the animals were placed on the instrument and fundus photographs were taken. Animals were then given intraperitoneal injection of fluorescein (AK-FLUOR 10%, Sigma Pharmaceuticals, North Liberty, IA, USA) and the retinal vasculature was imaged with blue light illumination after 5–8 min when all the vessels were filled.

### 2.5. Acellular Capillaries Quantification

Trypsin digestion of the retina was performed according to a previously published protocol [32,33]. Briefly, eyeballs were enucleated and incubated in 4% paraformaldehyde overnight. Retinas were isolated, washed, and digested in elastase solution (40 Units elastase/mL; Sigma-Aldrich, St. Louis, MO, USA) to remove the non-vascular tissue. The vascular beds were mounted on glass slides followed by staining with periodic acid–Schiff’s base and hematoxylin. About 5–6 fields from the central to mid-periphery were imaged and the number of acellular capillaries per square millimeter were quantified.

### 2.6. Immunohistochemistry

Immunohistochemical staining of mouse retinas was performed according to a previously published protocol [34]. Briefly, mice were euthanized and eyes were immediately enucleated and fixed in 4% paraformaldehyde (PFA) solution for 15 min. Cornea and lenses were carefully removed and posterior cups were incubated in 15% sucrose solution in phosphate-buffered saline (PBS) overnight at 4 °C after washing briefly in PBS. Posterior cups were transferred to 30% sucrose in PBS for 3–4 h, then embedded in optimal cutting temperature (O.C.T) medium and immediately frozen on dry ice. The frozen samples were stored at −80 °C until further processing. The sections were thawed at room temperature for 4 h, washed in PBS for 5 min, and permeabilized with 0.25% Triton-X in PBS for 5 min at room temperature. Sections were blocked with 10% horse serum in 1% bovine serum albumin (BSA) for 2 h then incubated with primary antibody diluted in blocking solution (1:100 dilution) overnight at 4 °C. The antibodies used were rabbit anti-GFAP (Abcam, MA, USA), mouse mAB HIF-1α antibody (Novus Biologicals, CO, USA), LXR-β polyclonal antibody (Invitrogen, IL, USA), rabbit anti-Vimentin (Cell Signaling Technology, MA, USA), and isolectin GS-IB4 Alexa Fluor 568 (Life Technologies, OR, USA). Sections were then washed and incubated in fluorescent-labeled secondary antibodies (goat anti-rabbit IgG Alexa Fluor 488, Life Technologies, OR, USA) for 1 h at room temperature, followed by washing and incubation with 4′,6-diamidino-2-phenylindole, dihydrochloride (DAPI) solution (Life Technologies, OR, USA) for 5 min at room temperature. Finally, sections were washed and mounted with anti-fade mounting medium (Vector Laboratories, CA, USA) for imaging. Image analysis was completed in a masked fashion using four images taken at defined positions and quantified using ImageJ software. The analysis was performed in a masked fashion by three separate observers, then averaged. To achieve unbiased results, positive and negative controls were included alongside experimental test and control groups. Fluorescent microscopy was performed by trained masked operators. To address selection bias in immunofluorescence, the entire areas of retinal cross-sections were imaged.

### 2.7. Statistics

All experiments were repeated at least 3 times. All data were assessed using one-way ANOVA. When the results were significant, we determined which means differed from each other using Tukey’s multiple-comparisons test. Results are expressed as mean ± standard error of the mean (SEM). Statistical analysis was performed using GraphPad Prism, with *p* < 0.05 considered statistically significant. Only significant comparisons are shown in the figures. All the examiners were blinded to the identities of the samples they were analyzing.

## 3. Results

### 3.1. HFD Mice Have Normal Glucose Levels but Are Insulin-Resistant

**Figure 1:**
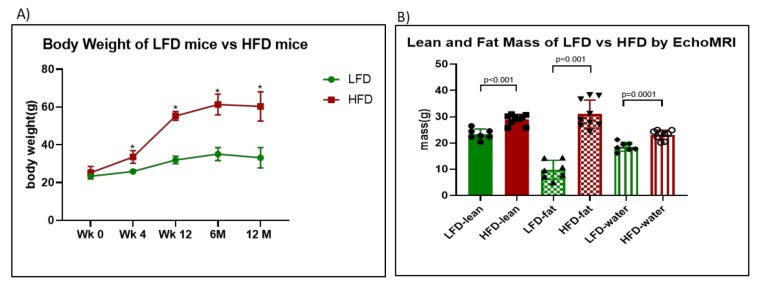

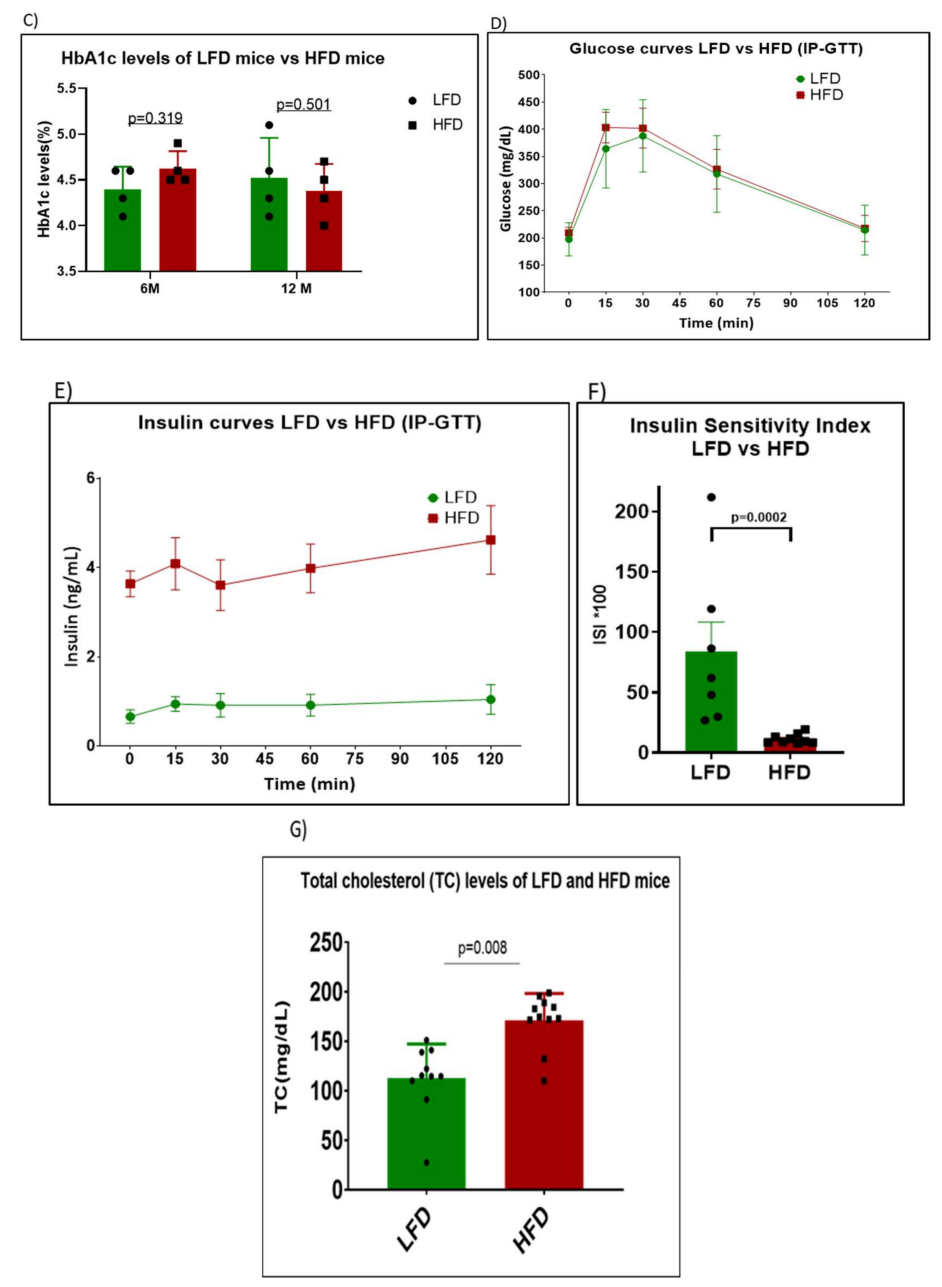
Body weight, glucose levels, and insulin sensitivity of high-fat diet (HFD) mice vs. low-fat diet (LFD) mice. (**A**) Body weights as measured for mice on LFD (green) and HFD (red) for 12 months. (* *p* < 0.000001; *n* = 6). (**B**) Lean mass, fat mass and water content of LFD mice vs. HFD mice. (**C**) Glycated hemoglobin (HbA1c) levels measured for the mice after 6 months and 12 months. (**D**–**G**) Glucose curves (*p* > 0.46 for all time points), insulin curves (*p* < 0.0018 for all time points), insulin sensitivity index, and total cholesterol levels for LFD mice vs. HFD mice, respectively, following intraperitoneal glucose tolerance test (IP-GTT) after 12 months of feeding.

We first sought to validate our model by confirming in our cohort that HFD feeding led to similar degrees of body weight gain as reported in the literature [10,35]. Mice on HFD showed increased body weights by 4 weeks of feeding (*p* = 0.0020). This increase was sustained throughout the 12-month observation period (Figure 1A). At 12 months, HFD mice had moderately higher lean mass (difference of 5.580 ± 1.003g, *p* < 0.0001) and water content (difference of 4.517 ± 0.876, *p* = 0.0001) but a markedly increased fat mass (difference of 21.15 ± 2.362, *p* < 0.0001) compared to LFD controls (Figure 1B). Unexpectedly, chronic high-fat feeding did not cause hyperglycemia. Despite feeding mice with a HFD for 12 months, the HbA1c levels were not different between HFD and LFD mice (Figure 1C). At 12 months, there was no difference in fasting blood glucose levels (Figure 1D, basal) and intraperitoneal glucose tolerance test (IP-GTT) did not show any significant differences between LFD and HFD mice (Figure 1D). However, due to the very high levels of insulin in HFD mice (0.6 ng/mL for LFD and 3.5 ng/mL for HFD; Figure 1E, basal), the insulin sensitivity index demonstrated that HFD mice had much lower insulin sensitivity compared to LFD mice (Figure 1E,F). Also, plasma total cholesterol levels were higher in HFD mice compared to LFD (174.4 vs. 114.9, *p* = 0.0008) (Figure 1G)

### 3.2. HFD Mice Have Functional Deficits in Their Retinas

**Figure 2:**
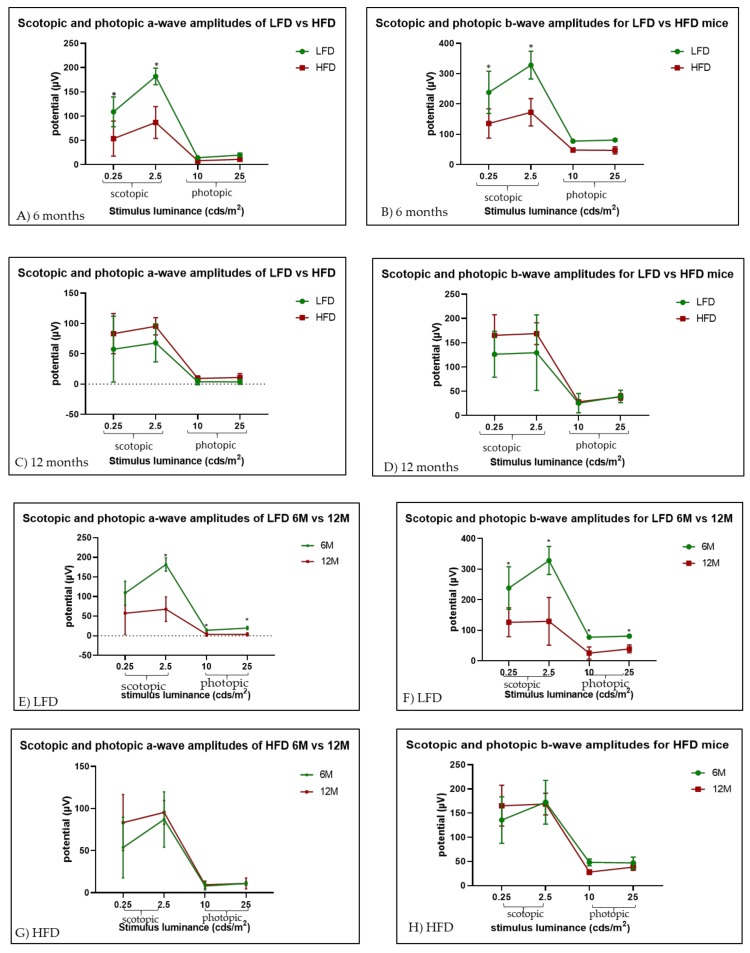
Assessment of retinal function of LFD mice versus HFD mice by electroretinogram (ERG). The amplitudes of a-waves and b-waves were assessed under both scotopic and photopic conditions for LFD mice and HFD mice after 6 months (**A**,**B**) and 12 months (**C**,**D**). LFD mice showed a significant reduction in retinal response between 6 months and 12 months of feeding (**E**,**F**), but HFD mice did not (**G**,**H**); (*n* = 4 for both groups).

Full-field ERG under both scotopic and photopic conditions was performed at 6 months and 12 months of HFD feeding (Figure 2A–D). HFD mice at 6 months showed significantly reduced a- and b-wave amplitudes under scotopic conditions (*p* = 0.00125 and *p* = 0.000002 for 0.25 cd.s/m^2^ and 2.5 cd.s/m^2^ stimulus luminance, respectively) but not photopic conditions when compared to LFD mice. After 12 months of feeding of the respective diets, the difference was not significant (*p* = 0.183 and *p* = 0.154 for 0.25 cd.s/m^2^ and 2.5 cd.s/m^2^ stimulus luminance respectively) (Figure 2C,D). Interestingly, when comparing 6 and 12 months of LFD feeding, the mice experienced marked reductions in both the a- and b- waves under both photopic and scotopic conditions at 12 months (Figure 2E,F), but no significant difference was noted in the HFD-fed mice (Figure 2G,H).

### 3.3. Fundus Photography shows Neural Retinal Lesions in HFD Mice

**Figure 3:**
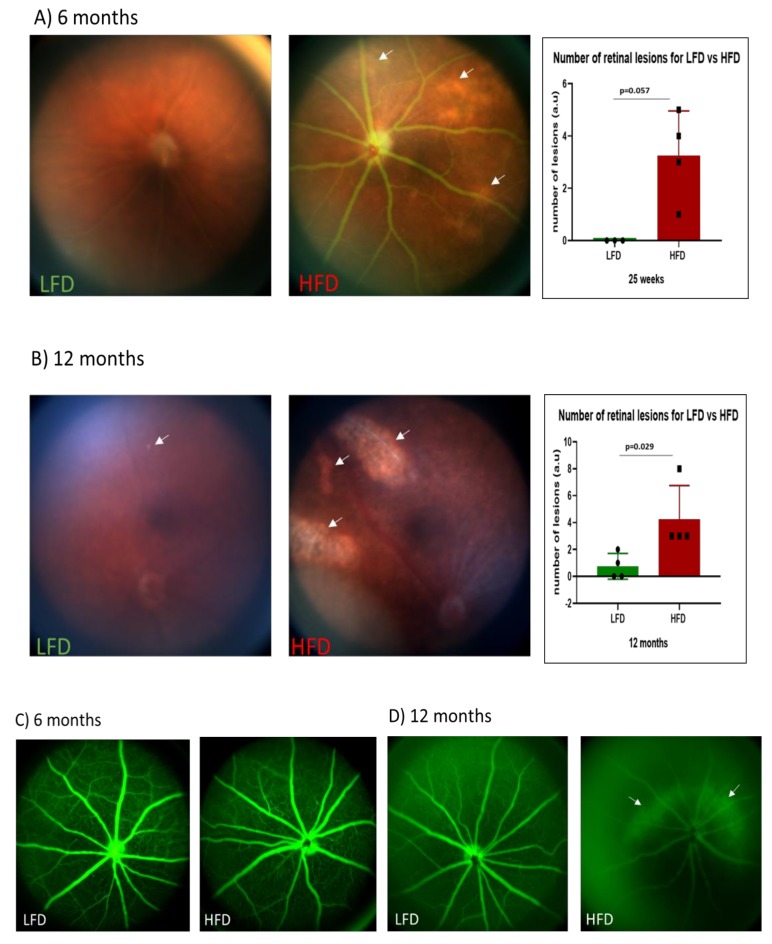
Assessment of retinal lesions by fundus photography (**A**,**B**) and vascular leakage by fluorescein angiography (**C**,**D**). HFD mice developed more neural infarcts ((**A**,**B**), white arrows) than LFD mice. No infarct was observed for LFD after 6 months (**A**). However, vascular leakage was observed in HFD mice after 12 months of feeding ((**D**), white arrows).

In humans, DR is associated with retinal lesions such as hemorrhages, microaneurysms, exudates, and “cotton wool spots” [36]. Fundus photography using Micron IV demonstrated retinal pathology in the HFD mice. Though not statistically significant, HFD mice showed a trend of increased numbers of “lipid-laden-like” lesions (Figure 3A) after 6 months (*p* = 0.057). However, with 12 months of feeding, HFD mice showed significantly higher number of lesions in the retina (Figure 3B).

### 3.4. Vascular Permeability Changes in HFD Mice

A hallmark of DR in humans is increased vascular permeability, ultimately leading to diabetic macular edema in humans. To determine if HFD mice developed a breakdown in the blood–retinal barrier, we assessed vascular leakage by fluorescein angiography (FA). At 6 months of HFD feeding, FA did not show any evidence of retinal vascular leakage and were similar to FAs in LFD controls (Figure 3C). However, after 12 months of HFD feeding, increased leakage of fluorescein was observed in the retina compared to LFD control retinas (Figure 3D).

### 3.5. Acellular Capillary Formation in HFD Mice

**Figure 4:**
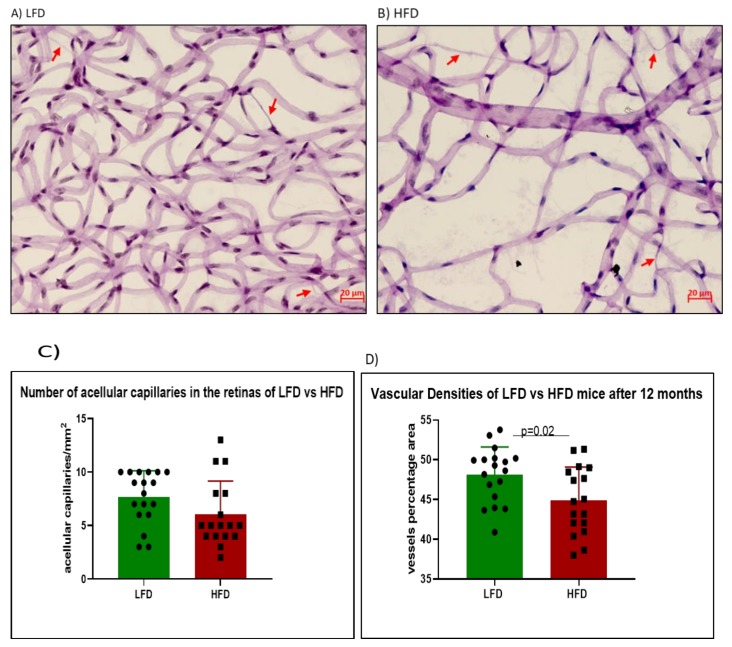
Enumeration of acellular capillaries in LFD and HFD mice after 12 months of feeding. Red arrows indicate acellular capillaries in the retinas of LFD (**A**) and HFD (**B**) mice. There was no significant difference in the number of acellular capillaries between both groups (**C**) (*p* = 0.086). However, HFD retinas showed lesser vascular densities compared to LFD retinas (**D**).

A well-established feature of diabetic microvascular dysfunction is an increase in the number of acellular capillaries in the retina, defined as basal membrane tubes lacking endothelial cells and pericyte nuclei. At 12 months of HFD feeding, there was no significant increase in acellular capillary numbers in the HFD mice (Figure 4B,C) compared to the LFD mice (Figure 4A,C). However, the HFD retinas showed lower vascular densities compared to LFD retinas (Figure 4D).

### 3.6. Retinal Damage, Hypoxia, and Lipid Transport in WD Mice

**Figure 5:**
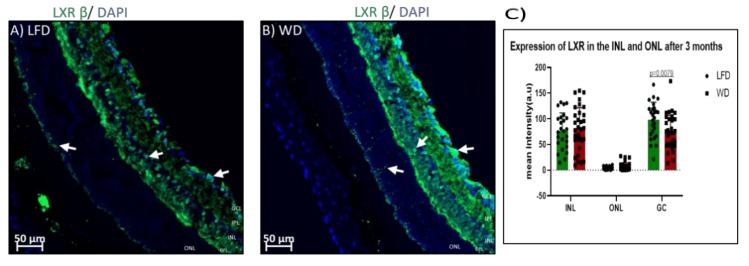

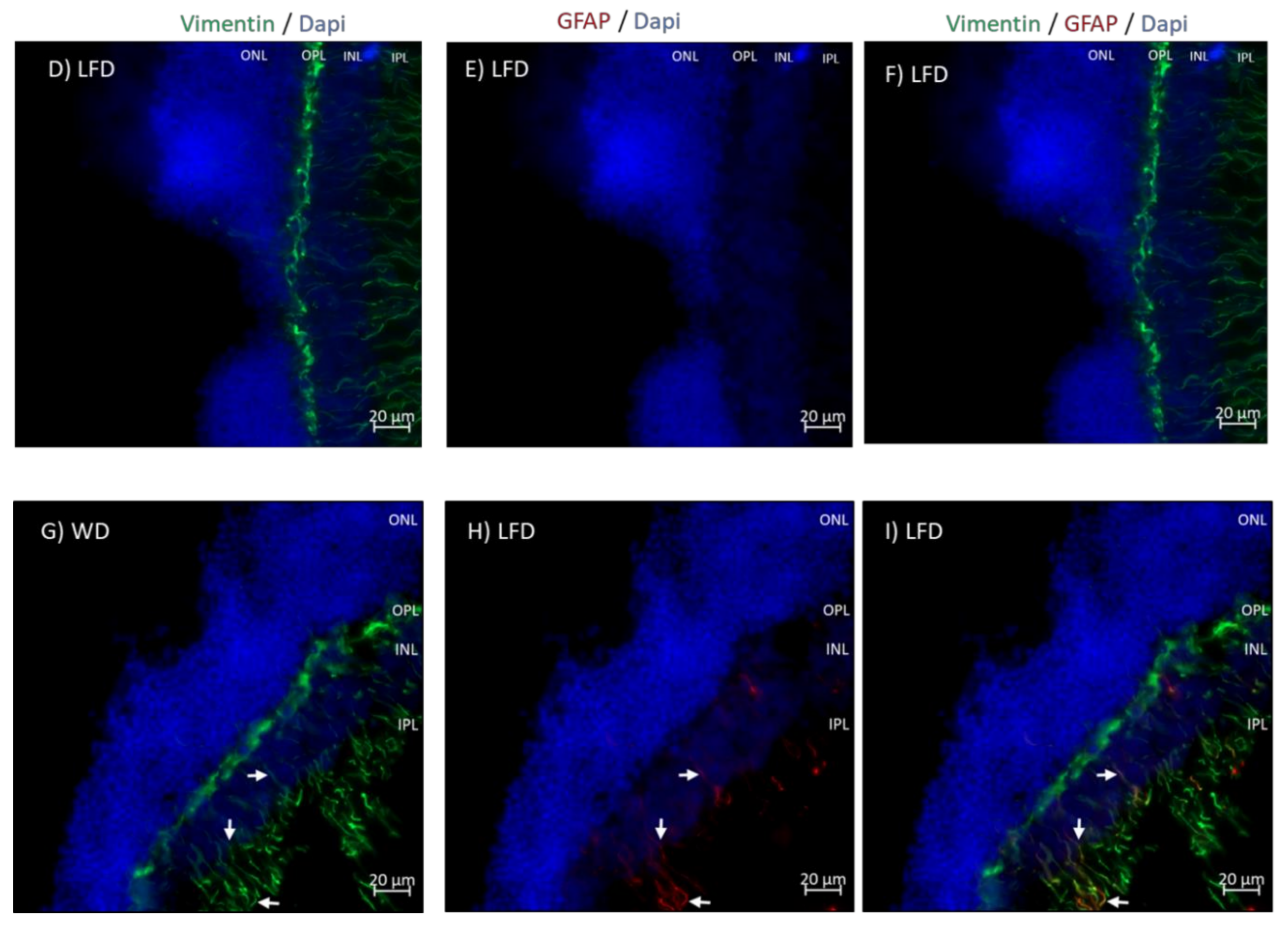
Retinal glial fibrillary acidic protein (GFAP) expression after 6 months of feeding. Some Muller cells in Western diet (WD) retinas express GFAP (**A**,**C**, white arrows), but not in LFD (**A**,**B**), indicating that the impact of WD is not uniform across all Muller cells. Co-localization with Vimentin, a known Mueller cell marker, showed increased expression of GFAP in some Mueller cells in WD mice (**G**–**I**) but not in LFD mice (**D**–**F**).

While the HFD represents a diet with 60% fat content that is used as a model of obesity and T2D, the WD with 40% fat content has garnered popularity as it represents a regimen closer to that actually ingested by humans. Since the WD diet has lower fat content and is not associated with hyperglycemia, we hypothesized that if retinal changes were present they would be subtle compared to those we observed with HFD feeding. To test the validity of our hypothesis, we performed IHC studies and first examined whether there was evidence of glial activation by examining expression of the glial marker GFAP after 6 months of WD feeding. Although there was no statistically significant difference (*p* = 0.88) in the total expression of GFAP between retinas of WD and LFD mice (Figure 5A–C), increased expression of GFAP was observed in selected Vimentin-positive Muller cells in the WD mice (Figure 5G–I) compared to LFD (Figure 5D–F). Increased expression of GFAP in Muller cells is supportive of increased oxidative stress and inflammation in these cells, and suggests that the impact of WD is not experienced uniformly across all Muller cells [37,38].

**Figure 6:**
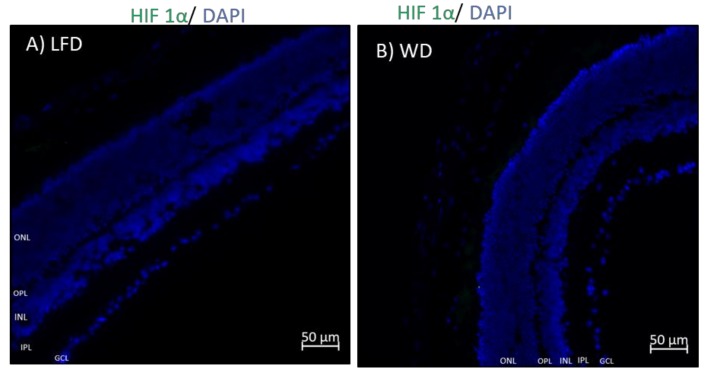

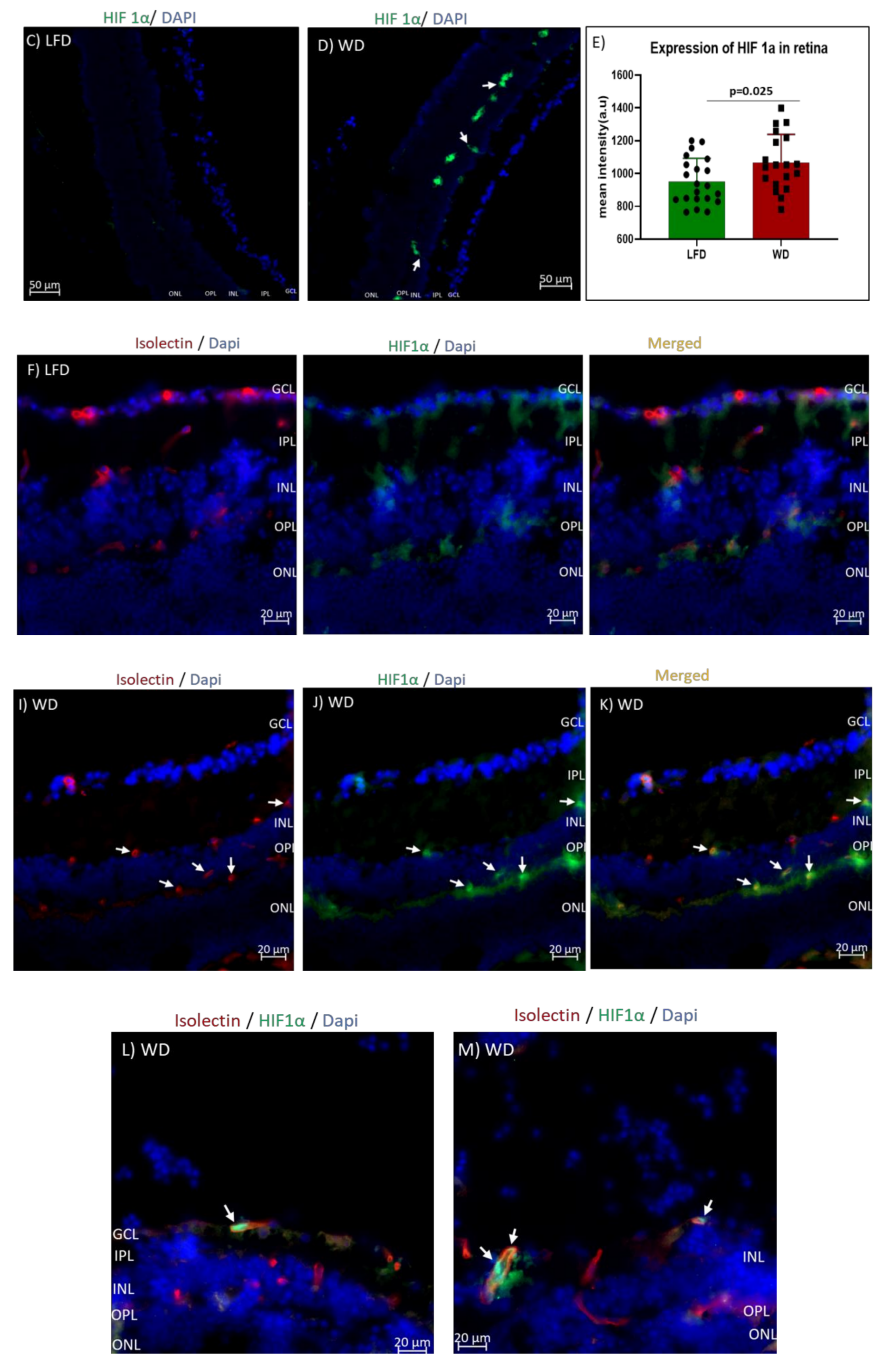
Retinal hypoxia-inducible factor 1 alpha (HIF-1α) expression after 3 and 6 months of WD feeding. There was increased expression of HIF-1α in WD retinas (**D**, white arrows) compared to LFD retinas (**C**), as shown by quantification (**E**). Also, there was no significant difference in expression of HIF-1α after 3 months of feeding (**A**,**B**). Co-localization with isolectin, a known vascular endothelial cell marker, showed increased expression of HIF-1α in some endothelial cells in WD mice (**I**–**K**) but not in LFD mice (**F**–**H**). (**L**,**M**) Magnified merged images from two different WD samples.

To assess whether WD feeding induced retinal hypoxia, changes in HIF-1α expression were examined by IHC. After 6 months of WD feeding, a significant increase (*p* = 0.025) in expression of HIF-1α was seen in WD mice (Figure 6D) compared to LFD mice (Figure 6C). This was not observed after 3 months of WD feeding (Figure 6A,B). Quantitation of HIF-1α expression is shown in Figure 6E, demonstrating that WD-fed mice exhibit higher levels than LFD-fed mice. Co-localization with isolectin, a known vascular endothelial cell marker, showed increased expression of HIF- 1α in some endothelial cells in WD mice (I–K) but not in LFD mice (F–H). Higher magnification images from two different WD samples are shown in Figure 6L,M.

**Figure 7:**
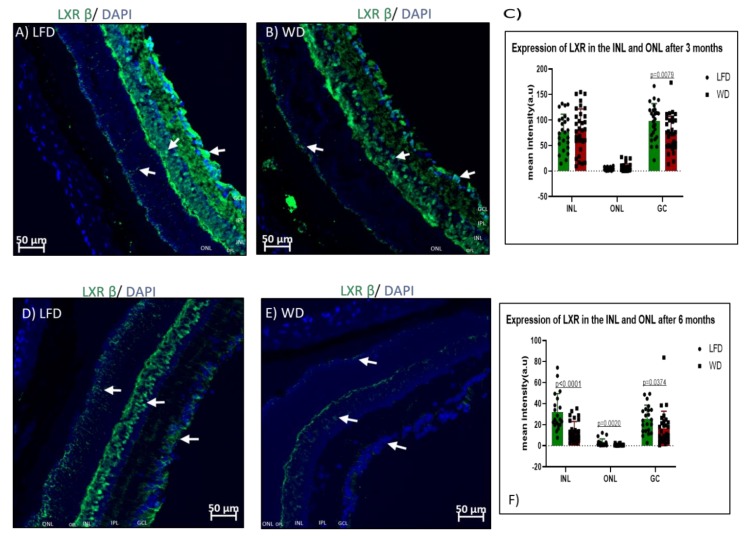
Retinal liver X receptor beta (LXRβ) expression after 3 and 6 months of feeding. After 3 months of either WD or LFD feeding, there was significant reduction in the expression of LXRβ in only the ganglion cell layer of WD mice (**B**) compared to LFD mice (**A**). However, after 6 months of feeding, there was reduced expression of LXRβ in the ganglion cell layer as well as inner and outer nuclear layers of WD mice (**E**, white arrows) compared to LFD mice (**D**). Quantification of LXR in the inner nuclear layer (INL) and outer nuclear layer (ONL) at 3 months shows reductions in the ganglion cell (GC) layer (**C**). At 6 months, reductions are seen in the INL, ONL, and ganglion cell (GC) layer of the WD-fed mice when compared to LFD mice.

Retinal lipid content is regulated in part by liver X receptor beta (LXRβ) expression. We next examined changes in LXRβ expression in the two experimental cohorts. In control mice, LXRβ localized predominantly in the ganglion cell layer, as well as the inner nuclear layer (Figure 7A), which is the location of the bipolar cells, horizontal cells, and amacrine cells. There was a significant reduction in expression of LXRβ in WD only in the ganglion cell layer (*p* = 0.0079) after 3 months of feeding (Figure 7B). However, after 6 months of WD feeding, WD mice (Figure 7E) showed significantly reduced expression of LXRβ in the ganglion cell layer (*p* = 0.0374), inner nuclear layer (*p <* 0.0001), and outer nuclear layer, as well as in the photoreceptors of the outer nuclear layer (*p* = 0.0020). The expression of LXRβ was reduced after 6 months compared to 3 months of feeding in both LFD (*p <* 0.0001) and WD (*p <* 0.0001) in the nuclear and ganglion cell layers, suggesting an age-related loss in LXRβ.

## 4. Discussion

Diabetic retinopathy causes both neural and vascular defects, with neural deficits preceding vascular changes [6,39,40,41,42]. Even before the onset of clinically detectable retinopathy, diabetic patients have a reduced ERG implicit time [43] and high-frequency flicker amplitude [44]. Later, they experience decreased vascular density [45]. In this study, we have shown that HFD feeding results in a suitable model of prediabetes, with the HFD cohort exhibiting insulin resistance and hypercholesterolemia without hyperglycemia. The retinopathy that is exhibited occurs over a slower time course than in T2D models, where both hyperglycemia and hyperinsulinemia exist.

The HFD mouse has previously been described as a model for T2D [7,46], as C57BL/6J mice fed HFD develop obesity and insulin resistance [47,48], but as we show in this study, this model has a distinct timeline and different characteristics than those seen in T2D. We show that HFD mice have hypercholesterolemia and insulin resistance but the absence of hyperglycemia, which is typical of T2D models.

In agreement with the literature, our study shows that mice fed a HFD have a sustained increase in body weight [6,49,50]. As confirmed by EchoMRI, the increase in body weight is primarily due to elevated body fat mass. After 12 weeks of feeding, HFD mice showed a two-fold increase in body fat mass over control LFD mice. Despite the marked increase in fat mass, HFD mice did not develop overt hyperglycemia. Glycated hemoglobin levels measured at 6 months and 12 months showed that both groups had normal glycated hemoglobin, thus indicating a key difference between the HFD model and other T2D rodent models, many of which are genetic. However, HFD mice develop hyperinsulinemia (Figure 1E,F), and their insulin production is sufficient to maintain euglycemia, as indicated by their glycated hemoglobin levels. The marked hyperinsulinemia we observed is supported by the literature [6,51,52,53,54]. In contrast, T2D in humans is characterized by not only insulin resistance but also the presence of sustained hyperglycemia and elevated HbA1c levels. When only insulin resistance is present, individuals are described as prediabetics [55,56].

Insulin resistance is believed to play a key role in diabetic neuropathy by increasing oxidative stress and mitochondrial dysfunction [57,58], and may also drive the early neural retinal dysfunction that we observed in our HFD mice. Thus, the HFD mice secrete sufficiently elevated insulin to maintain a normal glucose level, and as such the HFD model may be better characterized as a prediabetes model. Importantly, the incidence of prediabetes is often higher than that of diabetes [59]. The prevalence of prediabetes is also increasing; it is estimated that more than 470 million people worldwide will be suffering from prediabetes by 2030 [60]. Most importantly, the three classical microvascular complications, retinopathy, neuropathy, and nephropathy, have all been documented in individuals with prediabetes [61].

While classifications of diabetes remain “glucose-centric”, our study draws attention to the importance of earlier events, when glucose levels are still normal. Thus, in our model, hyperinsulinemia with hypercholesterolemia will likely lead to the retinal pathology observed. Not surprisingly, these pathologies take a longer time to develop than those typically seen when hyperglycemia is also present.

Systemic and retinal lipid abnormalities have been shown to promote retinal damage [16,62,63]. Previously, we demonstrated that diabetes-induced disruption of the LXR axis results in abnormal lipid metabolism, inadequate vascular repair, and localized and systemic inflammation [16,64]. The LXRs (LXRα and LXRβ) play important roles in cholesterol homeostasis [65]. They regulate the expression of reverse cholesterol transporters [12]. Activation of LXRs using pharmacological agents repress inflammatory genes such as TNF-α and IL-1β [66], inhibit the expression of pro-apoptotic factors [67], and prevent the development of DR [12]. We showed that use of GW3965, an LXR agonist, resulted in normalization of cholesterol homeostasis and repression of inflammatory genes, such as iNOS, IL-1β, ICAM-1, and CCL2 in the retina [16]. We found that inadequate cholesterol removal due to deficiency in LXR and reduced oxysterol production in the retina due to loss of cytochromes p450 27A1 and 46A1 resulted in widespread retinal pathology [68]. In the current study, we showed that concentrations of 40% fat in the diet were sufficient to reduce expression of LXR in the inner and outer nuclear layers.

Our study showed that HFD mice develop neural retinal deficits after 6 months of feeding, as both a-waves and b- waves were reduced under scotopic conditions. Unexpectedly, the a- and b- wave responses for LFD mice was significantly less after 12 months compared to the response after 6 months of feeding (*p <* 0.01 for both scotopic and photopic conditions), which suggests that the LFD may have detrimental effects on the neural retina. Because the composition of the diets must be isocaloric, when the amount of fat is reduced, some other dietary component needs to be increased to compensate. In the LFD, the amount of sucrose increases from 72 g to 354 g and 315 g of corn starch is also added so that the LFD can be isocaloric with the HFD. However, this largely occurs at the expense of making the diet high in carbohydrates. The literature supports that LFD may be detrimental [69,70,71]. While we were unable to find literature supporting the impact of LFD specifically on ERGs, the systemic consequences of LFD may indirectly affect the retina, for example by reduced availability of fat-soluble vitamins or changing retinal cholesterol metabolism. Moreover, the increased sucrose and cornstarch in the LFD may have direct deleterious effects [72,73]. LFDs promote insulin resistance, and while most of the research has been performed in humans, these findings may have relevance to murine studies. LFD, typically considered a high carbohydrate diet, is known to promote inflammation [74,75,76]. A recent study compared ERGs in HFD fed rats, Streptozotocin (STZ) rats and type 2 diabetes (T2D) rats at 6 months to controls. Kowluru found differences between the diabetic ERGs and controls, but no differences between the ERGs of the HFD rats compared to controls; however, Kowluru did not look at 12 month tests and the study was performed in rats, not in mice [77]. Thus, it is difficult to compare these findings with our results.

While neural damage was detected at 6 months, the vascular damage was not observed until much later. This is in agreement with Rajagopal et al. [6], who demonstrated that vascular damage was not observed at 6 months of HFD feeding. However, despite the absence of vascular damage after 6 months of HFD, we observed the presence of “lipid-laden like” lesions, and also neural infarcts similar to what is described in humans as “cotton-wool” spots. These lesions, which appeared to increase as the retinopathy progressed in the HFD mice, could become a useful measure of retinal damage and may be sensitive enough to use as a novel endpoint for the preclinical investigation of therapeutic agents.

GFAP is normally expressed in retinal astrocytes in rodents; however, during stress and inflammation, Muller cells [37] respond by increasing GFAP expression. In this study, we show that WD induces GFAP expression in selective Muller cells, supporting the presence of increased stress and inflammation in the retina of these mice. Kim et al. have reported increased inflammation in other tissues such as adipose tissue and intestines [78]. Lee et al. showed increased numbers of activated macrophages in the retina of HFD mice [79]. In both humans and rodents, obesity-induced diabetes is associated with hypoxia in tissues such adipose tissue, and suppression of HIF-1α mitigates tissue-specific pathological changes associated with HFD [80]. The liver, brain, kidney, and heart display tissue-specific regulation of HIF-1α under systemic hypoxia [81]. After 6 months, but not after 3 months, we observed that HIF-1α expression is increased in the WD retinas compared to LFD controls. Similar to our observation in the retina of 3-month-old mice on WD, Prasad et al. showed the absence of pimonidazole staining in the kidneys of 10–11-week old db/db mice [82], also indicating the absence of hypoxia response in the kidneys at this time point.

## 5. Conclusions

Our study demonstrates that HFD feeding generates a useful prediabetes model. Specifically, the combination of hypercholesterolemia and insulin resistance are sufficient to induce retinal dysfunction with a slower time course of development compared to T2D models such as the db/db mouse. In agreement with reports describing diabetes models, we show that neural functional deficits are the earliest indicator of damage in the retina of this prediabetes model before vascular changes. Key molecular targets such as HIF-1α and the LXRs provide insights into the retinal pathobiology observed in this hypercholesterolemic, hyperinsulinemic model. The appearance and frequency of neural infarcts or “lipid-laden lesions” in the retina of HFD mice could represent a novel endpoint for evaluation of therapeutic interventions.

## Supplementary Materials

The following are available online at https://www.mdpi.com/2073-4409/9/2/464/s1, Table S1: Detailed Composition of high-fat diet (HFD), Western diet (WD), and low-fat diet (LFD).
