# Supplementary material for: Characterizing the Retinal Phenotype in the High-Fat Diet and Western Diet Mouse Models of Prediabetes
Source: Cells. 2020 Feb 18;9(2):464. doi: 10.3390/cells9020464 (PMC7072836; doi:10.3390/cells9020464)
Supplement: Supplementary file 1 [file cells-09-00464-s001.pdf]

Supplementary Table 1. Detailed Composition of high-fat diet (HFD), Western diet (WD), and low-fat diet (LFD).

Class Description	Ingredient	Grams
**High-Fat Diet (HFD)**		
Protein	Casein, Lactic, 30 Mesh	200.00 g
Protein	Cystine, L	3.00 g
Carbohydrate	Lodex 10	125.00 g
Carbohydrate	Sucrose, Fine Granulated	72.80 g
Fiber	Solka Floc, FCC200	50.00 g
Fat	Lard	245.00 g
Fat	Soybean Oil, USP	25.00 g
Mineral	S10026B	50.00 g
Vitamin	Choline Bitartrate	2.00 g
Vitamin	V10001C	1.00 g
Dye	Dye, Blue FD&C #1, Alum. Lake 35-42%	0.05 g
**Low-Fat Diet (LFD)**		
Protein	Casein, Lactic, 30 Mesh	200.00 g
Protein	Cystine, L	3.00 g
Carbohydrate	Sucrose, Fine Granulated	354.00 g
Carbohydrate	Starch, Corn	315.00 g
Carbohydrate	Lodex 10	35.00 g
Fiber	Solka Floc, FCC200	50.00 g
Fat	Soybean Oil, USP	25.00 g
Fat	Lard	20.00 g
Mineral	S10026B	50.00 g

Vitamin	Choline Bitartrate	2.00 g
Vitamin	V10001C	1.00 g
Dye	Dye, Yellow FD&C #5, Alum. Lake 35-42%	0.05 g
**Western Diet (WD)**		
Protein	Methionine, DL	3.00 g
Carbohydrate	Sucrose, Fine Granulated	350.00 g
Carbohydrate	Lodex 10	100.00 g
Carbohydrate	Starch, Corn	50.00 g
Fiber	Solka Floc, FCC200	50.00 g
Fat	Butter, Anhydrous	200.00 g
Fat	Corn Oil	10.00 g
Mineral	S10001A	17.50 g
Mineral	Calcium Phosphate, Dibasic	17.50 g
Mineral	Calcium Carbonate, Light, USP	4.00 g
Vitamin	Choline Bitartrate	2.00 g
Vitamin	V10001C	1.00 g
Anti-oxidant	Ethoxyquin	0.04 g
Special	Cholesterol, NF	1.50 g
